# Spatial-Temporal Heterogeneity in Large Three-Dimensional Nanofibrillar Cellulose Hydrogel for Human Pluripotent Stem Cell Culture

**DOI:** 10.3390/gels9040324

**Published:** 2023-04-12

**Authors:** Jin Hao, Ying Chen, Mingjian Zhu, Yingqing Zhao, Kai Zhang, Xia Xu

**Affiliations:** 1 Biochemical Engineering Research Center, Anhui University of Technology, Ma’anshan 243002, China; 2 School of Chemistry and Chemical Engineering, Anhui University of Technology, Ma’anshan 243002, China

**Keywords:** human pluripotent stem cells, nanofibrillar cellulose hydrogel, 3D culture, pluripotency, local environment

## Abstract

One approach to cell expansion is to use large hydrogel for growing a large number of cells. Nanofibrillar cellulose (NFC) hydrogel has been used for human induced pluripotent stem cell (hiPSCs) expansion. However, little is known about the status of hiPSCs at the single cell level inside large NFC hydrogel during culture. To understand the effect of NFC hydrogel property on temporal–spatial heterogeneity, hiPSCs were cultured in 0.8 wt% NFC hydrogel with different thicknesses with the top surface exposed to the culture medium. The prepared hydrogel exhibits less restriction in mass transfer due to the presence of macropores and micropores interconnecting the macropores. More than 85% of cells at different depths survive after 5 days of culture inside 3.5 mm thick hydrogel. Biological compositions at different zones inside the NFC gel were examined over time at a single-cell level. A dramatic concentration gradient of growth factors estimated in the simulation along 3.5 mm NFC hydrogel could be a reason for the spatial–temporal heterogeneity in protein secondary structure and protein glycosylation and pluripotency loss at the bottom zone. pH change caused by the lactic acid accumulation over time leads to changes in cellulose charge and growth factor potential, probably another reason for the heterogeneity in biochemical compositions. This study may help to develop optimal conditions for producing high-quality hiPSCs in large nanofibrillar cellulose hydrogel at scale.

## 1. Introduction

Human pluripotent stem cells (hPSCs), with a capacity for unlimited proliferation and differentiation into almost all types of cells, have become potential cell sources for many applications, such as drug testing and regeneration medicine. In recent years, since stem cell therapy has significantly advanced, a critical challenge in this field is not only the large number but also the high quality of stem cells required for clinical applications and studies of disease mechanisms or drug screening.

Conventional two-dimensional (2D) culture used for stem cell expansion could lead to abnormal morphology and polarization [1,2], resulting in less stability and shorter lifespans [3], causing failure in the clinical trial due to unnatural microenvironment [4]. By contrast, 3D culture is much desired to manufacture hPSCs at scale since it provides a mimic environment for cells to communicate with surrounding cells and matrix to maintain cell functions in in vitro cell culture [5,6].

Hydrogels are three-dimensional networks of hydrophilic polymers which are attractive for cell expansion due to their biocompatibility, tunable mechanical properties, and ability to support cell growth and differentiation. Many attempts have been made to construct 3D hydrogel for hiPSC culture [7], such as enzyme-mediated hyaluronic acid–tyramine (HA–Tyr) hydrogel [8], thermo-responsive hydrogel based on hyaluronic acid, poly(N-isopropylacrylamide) [9], a hydrogel for scalable hPSC expansion [10,11,12], and modified PGMA preventing hPSC colonies stasis [13].

Stem cell expansion in hydrogels is a promising technology for tissue engineering and regenerative medicine. One approach is to encapsulate stem cells within smaller hydrogel microspheres or microparticles [14]. This approach allows for precise control over the size and distribution of stem cells within the hydrogel [15]. Another approach is to use large hydrogel, which can support the growth of a large number of cells. In vivo, the cells are exposed to a chemostatic environment due to a constant fresh nutrient supply and removal of metabolic results in waste via circulatory systems to support viable cell growth [16]. However, due to mass transfer limitation in large hydrogels, in vitro distribution of nutrients and accumulation of waste metabolites inside 3D scaffold culture are dependent on properties of 3D matrices, leading to various microenvironments around the cells, even causing cell death with increasing thickness of 3D matrices [17]. Hence, the biochemical condition for cell expansion in large hydrogels is a critical challenge to promote cell proliferation.

Hydrogels with features to facilitate the mass transport of nutrients, metabolic wastes, and soluble molecules are desired for cell expansion in a large hydrogel. Recently a novel plant-derived nanofibrillar cellulose (NFC) hydrogel has been demonstrated to maintain hPSC pluripotency [18]. To explore the suitability of large nanofibrillar cellulose hydrogel for hPSC culture, it is paramount to understand the spatial–temporal heterogeneity of cells inside the hydrogel. Herein, the property of nanofibrillar cellulose hydrogel was initially estimated. Cell morphology, viability, and pluripotency at different locations inside hydrogel were exploited. Biological compositions at a single-cell level were determined using synchrotron radiation-based Fourier transform infrared (SR-FTIR) spectroscopy. An in-depth analysis of the second derivative spectra by principal component analysis (PCA) was performed. Furthermore, the spatial distribution of nutrient supply and metabolic waste inside hydrogel was predicted over time using Comsol. 

## 2. Results and Discussion

### 2.1. Cellulose Characterization

The NFC we used here is a commercial product isolated from bleached birch pulp via a controlled homogenization process. The purified cellulose was diluted and sterilized with a resulting concentration of 1.7 wt%. The NFC hydrogel used in this study was prepared by mixing 1.7 wt% cellulose with a certain amount of PBS buffer and then settled down in the incubator at 37 °C before determining its property. 

**Figure 1:**
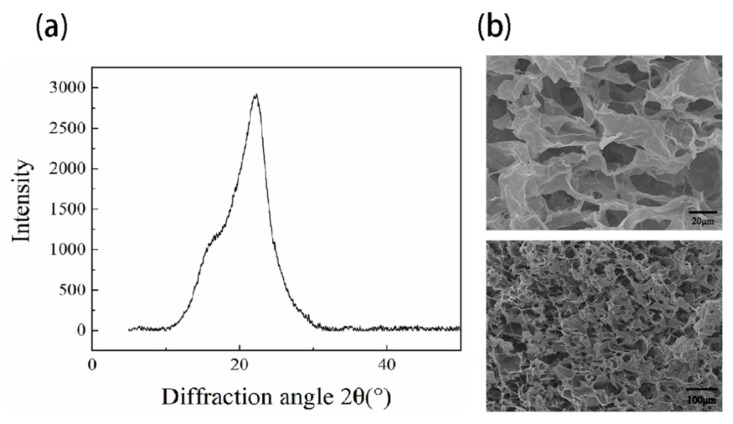
Characterization (**a**) XRD of Nanofibrillar cellulose (**b**) SEM images of NFC hydrogel (0.8 wt%) at different magnifications.

Initially, the cellulose was characterized using XRD and FTIR. XRD results in Figure 1a demonstrated that a strong peak at 2θ of 22.3° and a shoulder at 2θ of 15.8° associated with a crystalline peak at (2 0 0) and (1 1 0), respectively [19]. FTIR results (Figure S1) showed peaks at 3353 and 2905 cm^−1^ assigned to OH stretching, symmetrical and asymmetric stretching vibrations of CH, peaks at 1429 and 1370 cm^−1^ associated with CH_2_ bending and CH bending, peaks at 1318, 1040, and 895 cm^−1^ assigned to CH_2_ wagging, COH deformations and antisymmetric out-of-phase ring stretching, all corresponding to the vibration of cellulose-related chemical bonds [20,21]. The absence of a peak at 1726 cm^−1^ indicated no acetyl and aromatic ester groups of hemicellulose [22]. 

Hydrogel properties such as pore size and pore interconnectivity are critical for nutrient supply during 3D cell culture. Before we evaluated cell responses in 3D NFC hydrogel, NFC hydrogel at the concentration of 0.8 wt% (used for 3D cell culture) was characterized using SEM. As shown in Figure 1b, the hydrogel exhibited macropores (around 20–60 μm) and micropores (around 5 to 20 μm) interconnecting the macropores (Figure 1b), implicating less restriction in mass transfer. 

### 2.2. Cell Morphology and Cell Viability of Human iPSCs in 3D Culture

**Figure 2:**
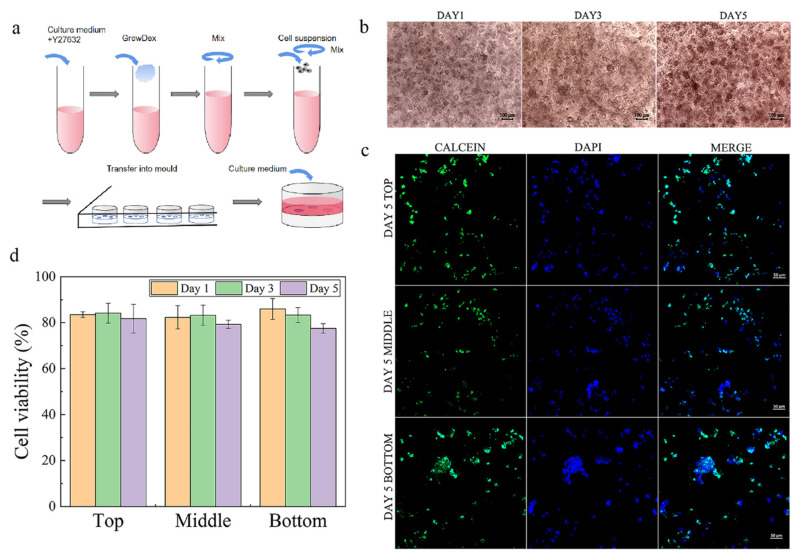
Cell morphology and viability after 5 days of culture. (**a**) Scheme for construction of NFC hydrogel embedded with hiPSCs. (**b**) Typical images for cell morphology on days 1, 3, and 5. (**c**) Typical images for cell viability at different zones at the end of 5 days of culture. (**d**) Cell viability at different zones on day 5. Error bars denote the means ± SEM.

The NFC was isolated from bleached birch pulp via a controlled homogenization process, then diluted at a concentration of 1.7 wt%. To construct NFC hydrogel with hiPSCs, the cells were mixed with the NFC at the final concentration of 0.5 and 0.8 wt%, then transferred to a culture mold (Figure 2a). Around 85% of cells survived during culture under both concentrations, but the hydrogel with 0.5 wt% was too fragile; it collapsed when changing the medium. Hence, the hydrogel with 0.8 wt% was chosen for hydrogel characterization and later experiments. Then, we examined the cell morphology in the hydrogel with different thicknesses of 2, 3.5, and 5 mm. The cell morphology in 2 mm hydrogel was similar to that in 3.5 mm, whereas the cell morphology in 5 mm hydrogel exhibited more compact and smaller (Figure S2). Therefore, the hydrogel with 3.5 mm thick was selected for further experiments to differentiate the heterogeneity inside NFC hydrogel during the following culture.

As shown in Figure 2b, the cells cultured in 3.5 mm thick hydrogel exhibited similar morphology, but the cell clump size became larger, indicating that a 3.5 mm thick NFC hydrogel is able to support colony formation of hiPSC, which is consistent with previous studies [11,18]. During culture, around 80% of cells at different zones (top zone: 0–0.5 mm, middle zone: 1.5–2 mm, bottom zone: 3–3.5 mm) remained alive on days 1, 3, and 5 (Figure 2c, Figures S3 and S4). This suggests that 3.5 mm thick nanofibrillar cellulose hydrogel can maintain cell survival and form a colony. 

### 2.3. Spatial–Temporal Biological Compositions of Cells during 3D Culture

**Figure 3:**
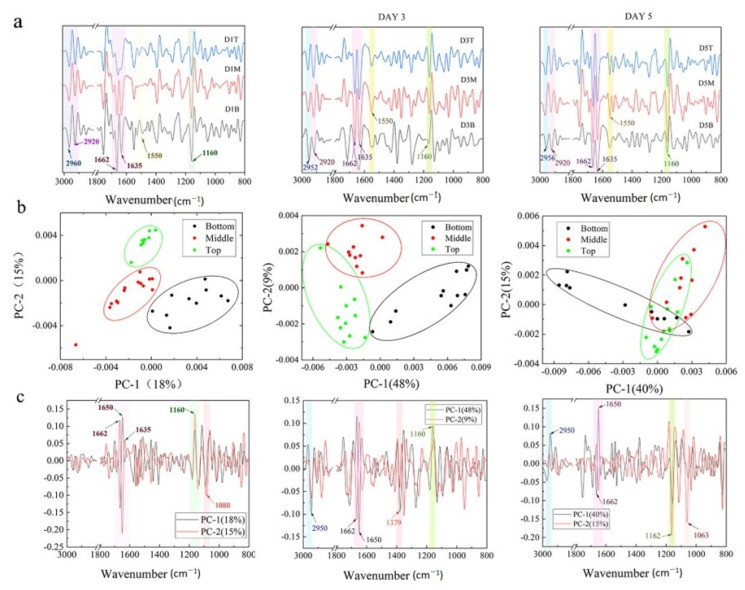
Average second derivative spectra and principal component analysis for cells during culture. (**a**) Average second derivative spectra of the cells at different zone on days 1, 3, and 5. (**b**) The representative score plots of the PCA of hPSCs at different zones in 3D hydrogel during culture on days 1, 3, and 5. (**c**) The loading plots of the PCA for hPSCs cultured on days 1, 3, and 5.

To explore the biological components of cells at the single-cell level, the SR-FTIR was used due to its higher resolution. The band assignment is listed in Table S2. To differentiate the nucleic acid, protein, and lipid alternation, the second derivative spectra were processed, and principal component analysis (PCA) was carried out. As seen in Figure 3a, the cells at different zones exhibited an intense νC–O and δC–O–H band at ~1160 cm^−1^, protein amide I band at ~1635, ~1650, ~1662 cm^−1^, an amide II band at ~1550 cm^−1^, a CH_3_ asymmetric stretching band at ~2960 cm^−1^, and a CH_2_ asymmetric band at ~2920 cm^−1^ [23].

The biological compositions of cells at different depths were further investigated in detail. On day 1, the cells at the bottom zone showed a significant difference from the top zone along PC2 (Figure 3b), interpreted by the strong loading ascribed to protein glycosylation (Figure 3c) [24] and secondary structure of the protein (Figure 3c and Figure S5). However, this PC only accounted for 15% of the variances, suggesting that the difference between the cells at deep, middle, and surface zones was not significant. On day 3, the difference between the top and middle zone disappeared while the cells at the bottom zone still exhibited a distinct separation from the cells at the top zone along PC1 (48%), implicating that the difference in biological compositions associating with 2950, 1662, 1650 cm^−1,^ and 1162 cm^−1^ (Figure 3b,c and Figure S5) was noticeable. Similar phenomena were observed for the cells on day 5. Some cells at the bottom zone obviously separated from the other cells along PC1 (40%) (Figure 3b), exhibiting the difference in the protein secondary structure and protein glycosylation, which could influence various biological phenomena [25,26].

### 2.4. Cell Pluripotency of hiPSCs in 3D Nanofibrillar Cellulose Hydrogel

**Figure 4:**
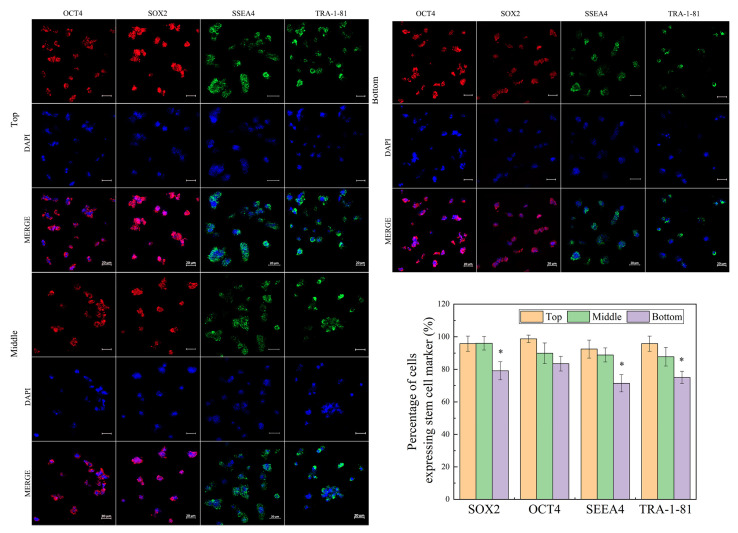
Human iPSC stemness at different locations on day 5 cultured in 3D hydrogel. Error bars denote the means ± SEM. Statistical analyses were performed by unpaired Student’s *t*-test (* *p* < 0.05).

We further examined the pluripotency of hiPSCs. Immunohistochemical analysis (Figure 4) indicated that hiPSCs at the top zone were strongly positive against stem cell markers expressed in non-differentiated ES cells (OCT4, SOX2, SSEA-4, and TRA-1–81) [17], whereas some cells at the bottom zone did not maintain undifferentiated status at the end of culture (Figure 4), which is consistent with results in Figure 3. This difference in pluripotency probably resulted from the low concentration of FGF2 and TGF-β1 at the bottom zone, which is lower than the concentration threshold for maintaining the cell pluripotency [27,28].

### 2.5. Spatial–Temporal Distribution of Solutes in 3D Hydrogel during Culture

To elucidate the local environment inside hydrogel, the solute distribution inside hydrogel was evaluated. Initially, the gel fiber radius was determined by evaluating the efficient diffusion coefficient of FITC-tagged dextran and FITC-tagged bovine serum albumin in 0.8 wt% nanofibrillar cellulose hydrogel (the dimension of 3D hydrogel is Ø5 mm × 3.5 mm) based on their concentration change over time (Figure S6). Then, the gel fiber radius of 0.326 nm was obtained using Equations (1) and (2) [29]. Furthermore, the efficient diffusion coefficients of glucose, lactic acid, and growth factors of TGF-β1 and FGF2 were derived, as listed in Table 1. 

**Figure 5:**
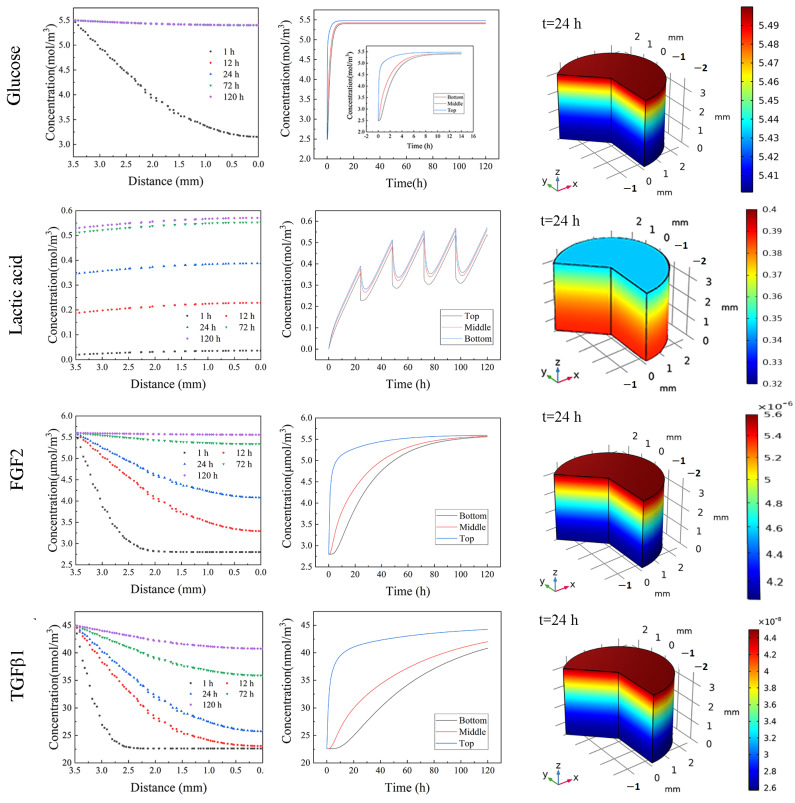
Solute distribution inside 3D hydrogel at different times and locations.

Since the hydrogel was restricted in a mold, the mass transfer was considered to be in the axial direction. As shown in Figure 5, glucose exhibited uniform distribution even on day 1. By contrast, FGF2 and TGF-β1 showed a remarkable concentration gradient from the bottom to the top inside the 3D hydrogel, indicating that the cells at the deep zoom were exposed to less FGF concentration until day 3. TGF-β1 is an important factor for maintaining undifferentiation. The simulation results revealed a dramatic difference in the TGF-β1 concentration between the bottom zone and the other zones even from day 1, and this gap became even worse with the increase in culture time (Figure S7). The experimental results (Figure 4) and simulation results together implicate that hiPSCs experience differentiation at the bottom zone during a 3D culture in large NFC hydrogel due to low TGF--β1 concentration. For future study, the differentiation potential of hiPSCs from different locations in large NFC hydrogel should be revealed.

Due to the continuous production of lactic acid during culture, lactic acid showed a steadily increasing concentration before changing culture medium, then dropped, followed by a further increase, suggesting that the cells experienced a more acidic culture environment over time, causing the change in charge of hydrogel and growth factors and further affecting the interaction among the hydrogel, growth factors, and cells. All these together resulted in the difference in glycosylation and protein production for the cells at different zones, further affecting the undifferentiated status at the bottom zone (Figure 4). Moreover, due to the lactic acid accumulation over time, the cells were exposed to a much more acidic culture environment, causing a change in the potential of protein and nanofibril cellulose, leading to the change in the interaction of cells with growth factor and hydrogel, further affecting the interaction of cells with the local environment, eventually influencing the undifferentiated status of cells [30]. It should be pointed out that in comparison with a restricted mold, exposing the hydrogel to culture medium on all sides would improve the nutrient supply and efficiently remove waste, leading to less spatial–temporal heterogeneity in growth factors distribution and less waste accumulation, indicating that hPSC could be expanded in 3D nanofibrillar cellulose hydrogel with a larger dimension.

## 3. Conclusions

The spatial–temporal heterogeneity of hPSCs cultured in a large 3D nanofibrillar cellulose hydrogel was explored. TGF-β1 and FGF2 exhibit a significant concentration gradient throughout the hydrogel, and the metabolic product of lactic acid gradually builds up over time, leading to a more acidic environment and less negatively charged nanofibrillar cellulose hydrogel, resulting in changes in the potential growth factors, further affecting the interaction of cells with the environment, and eventually causing some cells at the deep zone losing undifferentiated status. The heterogeneity can be reduced by exposing the cell-embedded hydrogel to an open environment. Hence, nanofibrillar cellulose hydrogel with a larger dimension could be a platform for hiPSCs’ scalable expansion. 

## 4. Materials and Methods

### 4.1. Materials

Nanofibrillar cellulose (1.5% (*w*/*v*)) was purchased from UPM Biomedical, Helsink, Finland. Human iPSCs, line SiDSH, were obtained from Sidansai Biotec (Shanghia, China). Polystyrene with MW of 8400, 33,400, 101,300, 171,000, and 311,300 g/mol (analytical standard), Triton X-100 (laboratory grade), calcein-AM, and bovine serum albumin (BSA, 98%) were purchased from Sigma (Saint Louis, MO, USA). FITC-dextran (5 kDa) and FITC-BSA (98%) were obtained from Solarbio (Beijing, China). NaCl (99.8%), KCl (GR, 99.8%), Na_2_HPO_4_·12H_2_O (99%), and KH_2_PO_4_ (99.5%) were purchased from Macklin (Shanghia, China). ROCK inhibitor Y-27632 was from APExBIO (Houston, TX, USA). TrypLE^M^ and PSCeasy^®^ human pluripotent stem cell medium were purchased from Gibco (Paisley, UK) and Cellapybio (Beijing, China), respectively. Matrigel and primary antibodies, rabbit anti-SOX2 polyclonal antibody, rabbit anti-Oct4 polyclonal antibody, mouse anti-TRA-1-81, and mouse anti-SSEA4 monoclonal antibody (all at 0.5 mg/mL) were purchased from BD Biosciences (San Jose, CA, USA). DAPI was purchased from Biyuntian (Shanghai, China). Normal donkey serum, Alexa Fluor 647 dye conjugated goat anti-rabbit IgG, Alexa Fluor 488 dye conjugated goat anti-mouse IgM, Alexa Fluor 647 dye conjugated goat anti-rabbit IgG, and Alexa Fluor 488 dye conjugated goat anti-mouse IgM (all >95%) were purchased from Abbkine (Wuhan, China). BaF_2_ optical windows were obtained from Ruipu (Wenzhou, China). OCT^TM^ tissue freezing medium and SYLGARD 184 Silicone Elastomer Kit (2%) were purchased from Leika (Vale of Glamorgan, UK), respectively. 

### 4.2. Cell Culture

The maintenance culture of hiPSCs cells was carried out using a feeder-independent culture method. Briefly, hiPSCs were cultured on Matrigel-coated plates at the dilution factor of 1:100, in PSCeasy^®^ human pluripotent stem cell medium at 37 °C and under 5% CO_2_. The culture medium was completely changed daily. After 5–7 days of culture, colonies were detached by TrypLE^TM^ at 37 °C for 5–7 min and then transferred to Matrigel-coated plates with 10 μM ROCK inhibitor Y-27632 for passage. Molds for 3D hydrogel culture were prepared using Sylgard 184 according to the manufacturer’s directions by mixing base and curing agent at 10:1. After defoaming, the PDMS was poured into 6-well with embedded cylinders (5 mm in diameter and different heights, 2, 3.5, and 5 mm) and cured at 65 °C for 12–24 h. Then, the embedded cylinders were removed. The molds were cleaned using DI water and dry air and then sterilized in the autoclave. For 3D culture, detached colonies were mixed with NFC at different concentrations of 0.8 wt%, transferred into a mold to form 3D matrices embedded with cells, and then cultured for 5 days. During culture, the culture medium was changed daily.

### 4.3. Characterization of 3D Hydrogel

The nanofibers are mainly composed of cellulose macromolecules with hemicellulose xylene forming dangling chains on the fibrillar surfaces. Initially, the molecular weight of nanofibrillar cellulose was determined. The crystalline phases present in nanofibrillar cellulose were determined using X-Ray diffraction (XRD, XRD-6000, Shimadzu, Kyoto, Japan) with Cu Kα radiation at 30 kV and 20 mA. Diffractograms were continuously collected at a diffraction angle from 2θ = 0° to 90° at a scan speed of 0.01 θ° s^−1^. The collected data was analyzed using Jade 6.5 software via 13-point smoothing with automatic baseline calibration. The functional groups in cellulose were further identified using Fourier transform infrared spectroscopy (FTIR, Nicolet IS10, Thermo-Fisher, Waltham, MA, USA). To look into the structure of 3D nanofibrillar hydrogel, the hydrogel settled into a mold, freeze-dried using a freeze-drier (FD5-3, Gold-SIM, Miami, FL, USA), then attached to an aluminum specimen holder using conductive adhesive tape, and covered with a thin platinum film. The samples were observed using a scanning electron microscope (SEM, NANO SEM 430, FEI, Columbia, SC, USA) at 50 eV–15 keV with Everhart–Thornley Detector (ETD). 

### 4.4. Cell Viability

To assess cell viability, the cells in 3D matrices were incubated with 1 μM Calcein-AM for 30 min at 4 °C in the dark and then washed with PBS. To visualize, the 3D matrices after staining were cryo-sectioned. The slice was mounted on the slide with DAPI and observed under a confocal microscope (LSM-800, Zeiss, Aalen, Germany). The cell viability was determined by counting living cells and total cells.

### 4.5. Immunohistochemistry 

Immunostaining was performed to detect the stem cell markers for hiPSCs during 5 days of culture. Briefly, the hydrogel with cells during culture was frozen in OCT^TM^. The frozen sections were cut into 15 μm thick slices using a cryostat (Leika, Germany) and then transferred to a glass slide. The slices were incubated with 4% (*v*/*v*) paraformaldehyde for 30 min at room temperature and then permeabilized with 0.5% (*v*/*v*) Triton X-100 in PBS for 30 min at room temperature, washed with PBS, permeabilized and blocked with 0.1% (*v*/*v*) Triton X-100, 1 wt% BSA, and 10% (*v*/*v*) normal donkey serum in PBS for 45 min at room temperature. In contrast, no permeabilization was performed for surface markers. Primary antibodies, rabbit anti-Sox2 polyclonal antibody, rabbit anti-Oct4 polyclonal antibody, mouse anti-Tra-1-81, and mouse anti-Ssea4 monoclonal antibody, were added to the cells at the final concentration of 10 μg/mL in PBS at 4 °C. Cells were then washed three times with 1 wt% BSA in PBS. Secondary antibodies, Alexa Fluor 647 dye-conjugated goat anti-rabbit IgG and Alexa Fluor 488 dye-conjugated goat anti-mouse IgM were diluted at 1:100 in PBS containing 1 wt% BSA. The diluted secondary antibody was applied to the cells for 120 min at room temperature in the dark. Cells were then washed with PBS three times and mounted with the DAPI. In all immunohistochemical analyses, sections from different culture time points were processed in triplicate. The 2D images of immunostained sections were obtained by a confocal laser-scanning microscope (LSM 710, Zeiss, Germany). The percentage of cells with pluripotency was determined by counting cells with stem cell markers and the total of cells. 

### 4.6. SR-FTIR 

To differentiate the biochemical composition at different locations inside hydrogel, the biochemical constituents were determined using synchrotron radiation-based Fourier transform infrared (SR-FTIR) spectroscopy at a single cell level [31,32,33,34,35,36,37,38] rather than the extraction from the large numbers of cells [39]. For spectroscopic measurements, the 3D matrices with cells were incubated with 4% paraformaldehyde for 60 min at room temperature to preserve the structural cellular components and to avoid disorder of internal structures and delocalization of biochemical constituents of cells. After three PBS washes, the hydrogel was cryo-sectioned as described above; then, the slices were transferred to BaF_2_ optical windows. The SR-FTIR measurements were carried out at the SR-FTIR spectroscopy station of BL01B beamline at the National Synchrotron Radiation Facility (Hefei, China), which was equipped with Bruker vertex 70 v Fourier transformation spectrometer, Bruker Hyperson 3000 microscope (Germany), and 64 × 64 elements Focal Plane Array (FPA) [40]. To achieve comparable signal intensities and signal/noise ratio, the aperture size of 20 × 20 μm was used throughout the experiment to collect spectra for the single cells. The signals of raw spectra were recorded in transmission mode between 650 cm^−1^ and 4000 cm^−1^, with 256 scans at 4 cm^−1^ resolution on the BaF_2_ substrate. The background was collected through a blank substrate. The total absorbance cartogram was generated by integrating within specific bands assigned to nucleic acids, proteins, and lipids, listed in Table S1. All data were smoothed (13-point) and baseline corrected. The OPUS 7.5 (Bruker, Coventry, UK) software package was used for data analysis. 

### 4.7. Solute Distribution within 3D Hydrogel

To further estimate the solute transport in a 3D hydrogel, the efficient diffusion coefficient was evaluated by monitoring the solute concentration of FITC-dextran 5 kDa and FITC-Bovine Serum Albumin (BSA). Briefly, cellulose hydrogel with a certain concentration was placed into a well in a 96-well black microplate with a transparent bottom, then the change of FITC-dextran/FITC-BSA concentration was monitored with time using a microplate reader (SynergyH1M, BIOTEK, Santa Clara, CA, USA) under static condition. BSA exhibits negative potential at the physiological condition. Hence, BSA adsorption on the cellulose hydrogel was neglected [41]. The effective diffusion coefficient in gel, *D_eff_*, is related to hydrogel property, based on the obstruction theory (Equations (1) and (2)) [42]. The solute diffusion coefficient inside hydrogel can be derived from Equations (3) and (4).

(1) $$D_{i,eff}=D_{0}exp(-0.84\alpha^{1.09})$$

(2) $$\alpha =\varphi ({\frac{r_{s+}r_{f}}{r_{f}})}^{2}$$

(3) $$N_{i}=-D_{i,eff}\nabla C_{i}$$

(4) $$N_{i}=(C_{media}-C_{i})\sqrt{\frac{D_{i,eff}}{\pi t}}$$

Here, *D_i,eff_* is the diffusion coefficient (m^2^/s), *φ* is the volume fraction, *r_f_* is the gel fiber radius (nm), *r_s_* is the radius of the diffusing particle (nm), *C_media_* and *C_i_* are the solutes *i* concentration in media and in gel, respectively (M), and *t* is the time (s). 

A one-dimensional diffusion–reaction model was applied to describe the solute distribution in 3D hydrogel during culture, given by Equation (5).

(5) $$\frac{{\partial C}_{i}(x,t)}{\partial t}=-D_{i,eff}\frac{\partial^{2}C_{i}\left(x,t\right)}{+{\partial x}^{2}}+R_{i}$$

Initial condition: *t* = 0: *C*(*x*, 0) = *C_0_* for *x* > 0; *C*(*x*, 0) = *C_media_* for *x* = h (the distance from the surface to the bottom of the gel). Boundary condition: *x* = 0: $\frac{\partial (x,t)}{\partial x}=0$.

Here, *x* is the distance between the bottom and position interested in the hydrogel (m), *R_i_* nutrient consumption rate, and metabolic production rate (mol s^−1^ m^−3^).

Solute distribution within 3D hydrogel was simulated using the COMSOL Multiphysics software (V 5.3) (Stockholm, Sweden). To simplify the process, it was assumed that no adsorption of solute molecules takes place due to the low density of the—COOH group within 0.8 wt% hydrogel, and the cell number was assumed to not change during culture. Fresh medium was applied during the simulation since the culture medium was changed daily during culture. Here, we simply took into account the glucose uptake and lactic acid production at a rate of 1.108 × 10^−5^ mol s^−1^ m^−3^ by cells [43] without considering TGF-β1 and FGF2 consumption/denaturation. Although at physiological pH conditions, FGF and TGF-β1 absorb on hydrogel due to interaction with cellulose via electrostatic interaction, the FGF2/TGF-β1 adsorption was neglected during simulation due to the low density of the —COOH group in nanofibrillar cellulose (only around 1%). The parameters used in the simulations are listed in Table S1. 

### 4.8. Statistic Analysis

All experiments were carried out in triplicate. Data are presented as the means ± standard error of the mean (SEM). Unpaired Student’s *t*-test was used to determine the differences between the two groups. *p* < 0.05 was considered statistically significant. Principle component analysis was performed for SR-FTIR data analysis. 

## Figures and Tables

**Table 1:** Effective diffusion coefficients of solutes and solute radius.

	Dextran	BSA	Glucose	Lactic Acid	FGF2	TGF-β1
*r_s_* (nm)	2.45	3.63	0.36	0.23	1.89	2.93
*D_0_* (×10^−10^ m^2^/s)	1.25	0.53	7.00	11.00	0.65	0.42
*D_eff_* (×10^−10^ m^2^/s)	0.79	0.196	6.84	10.85	0.488	0.22

## Data Availability

Data is contained within the article or supplementary material.

## Supplementary Materials

The following supporting information can be downloaded at: https://www.mdpi.com/article/10.3390/gels9040324/s1, Figure S1: FTIR for cellulose; Figure S2: Cell morphology in hydrogel with different thickness; Figure S3: Cell viability after 1 day of culture; Figure S4: Cell viability after 3 day of culture; Figure S5: Score and loading plots of PCA at the different zones; Figure S6 BSA and Dextran concentration change with time; Figure S7: Solute distribution in hydrogel at 72 and 120 h; Table S1: Global parameters and variables for the COMSOL simulation; Table S2: FTIR band assignment.
