# Supplementary material for: Spatial-Temporal Heterogeneity in Large Three-Dimensional Nanofibrillar Cellulose Hydrogel for Human Pluripotent Stem Cell Culture
Source: Gels. 2023 Apr 12;9(4):324. doi: 10.3390/gels9040324 (PMC10138276; doi:10.3390/gels9040324)
Supplement: Supplementary file 1 [file gels-09-00324-s001.zip › gels-2320401-supplementary.pdf]

Article

Spatial-Temporal Heterogeneity in Large Three-Dimensional Nanofibrillar Cellulose Hydrogel for Human Pluripotent Stem Cell Culture

Jin Hao [1,2,†], Ying Chen [1,2,†], Mingjian Zhu [1,2], Yingqing Zhao [1,2], Kai Zhang [1,2] and Xia Xu [1,2,*]

[1] Biochemical Engineering Research Center, Anhui University of Technology, Ma'anshan 243002, China
[2] School of Chemistry and Chemical Engineering, Anhui University of Technology, Ma'anshan 243002, China
* Correspondence: xiax@hotmail.com
† These authors contributed equally to this work.

Table of Contents

Figure S1 FTIR for cellulose 1
Figure S2 Cell morphology in hydrogel with different thickness……….2
Figure S3 Cell viability after 1 day of culture 3
Figure S4 Cell viability after 3 day of culture 4
Figure S5 Score and loading plots of PCA at the different zones 5
Figure S6 BSA and Dextran concentration change with time........ 6
Figure S7 Solute distribution in hydrogel at 72 and 120 h………. 7
Table S1 Global parameters and variables for the COMSOL simulation…........8
Table S2 FTIR band assignment........ 9

FTIR for cellulose

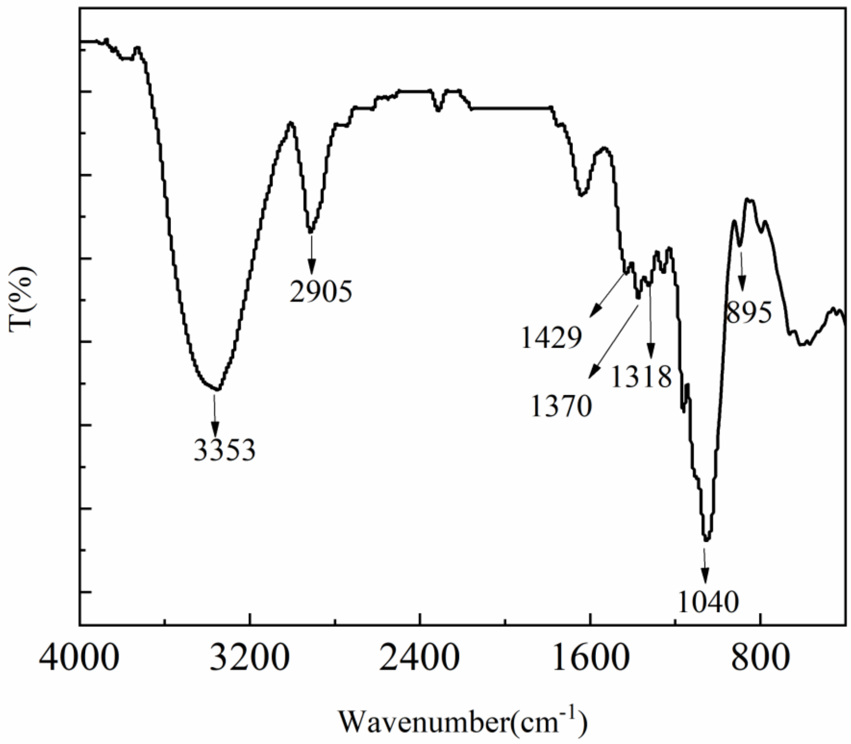

Figure S1. FTIR for cellulose.

Cell morphology in hydrogel with different thickness

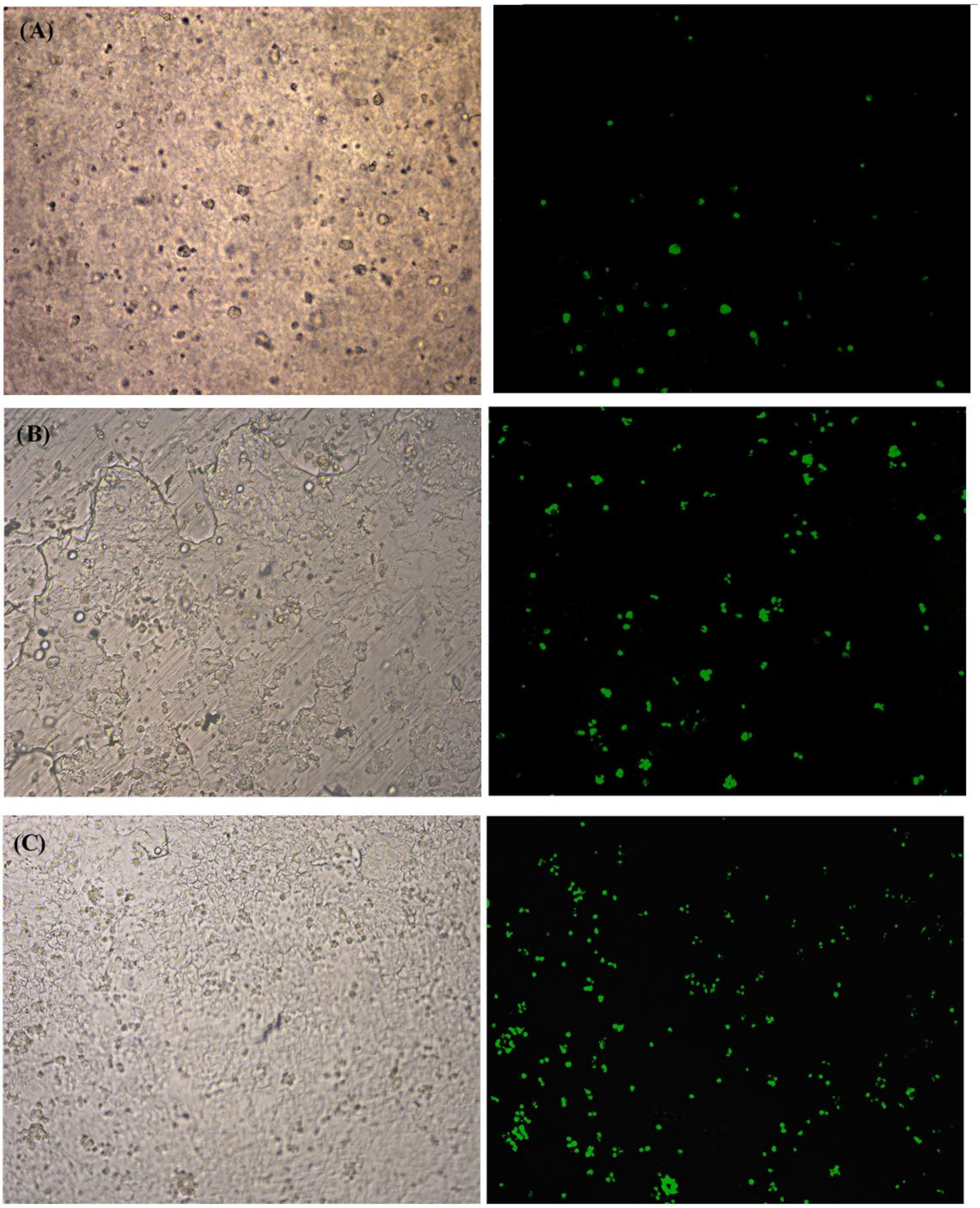

Figure S2. Cell morphology at day1 (A) 2mm (B) 3.5 mm (C) 5mm.

Cell viability after 1 day of culture

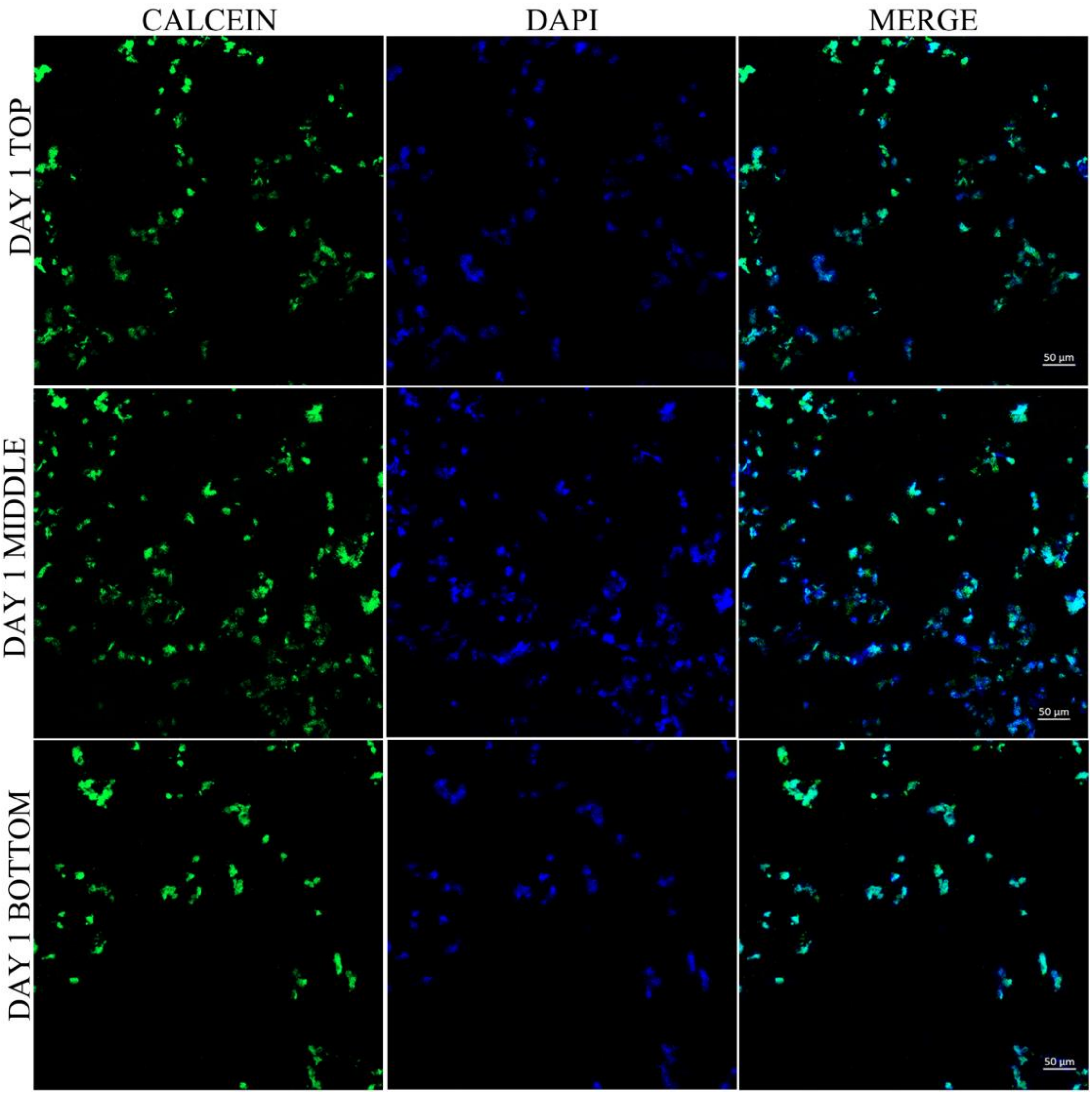

Figure S3. Cell viability at different locations after 1 day of culture.

Cell viability after 3 day of culture

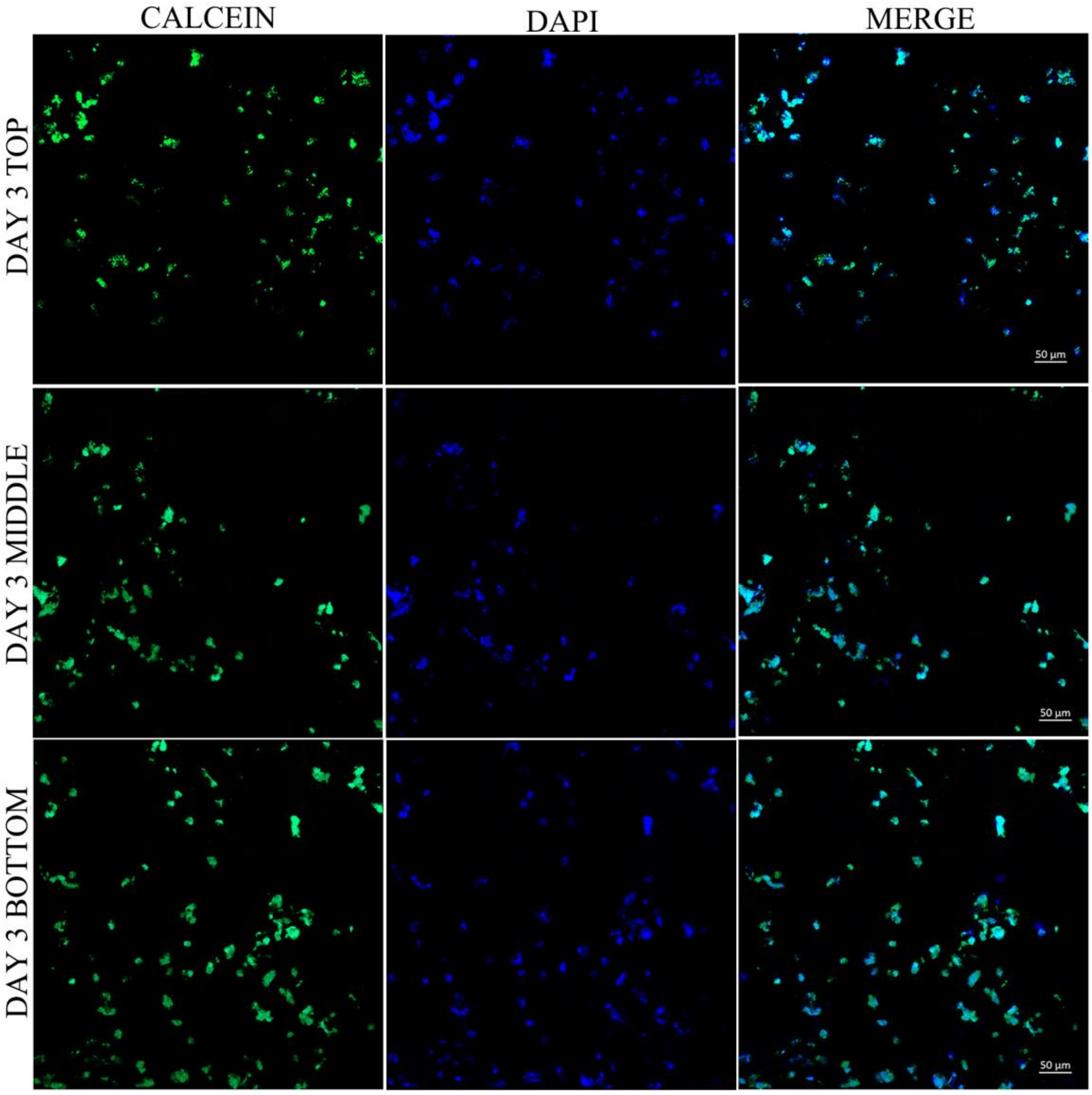

Figure S4. Cell viability after 3 days of culture.

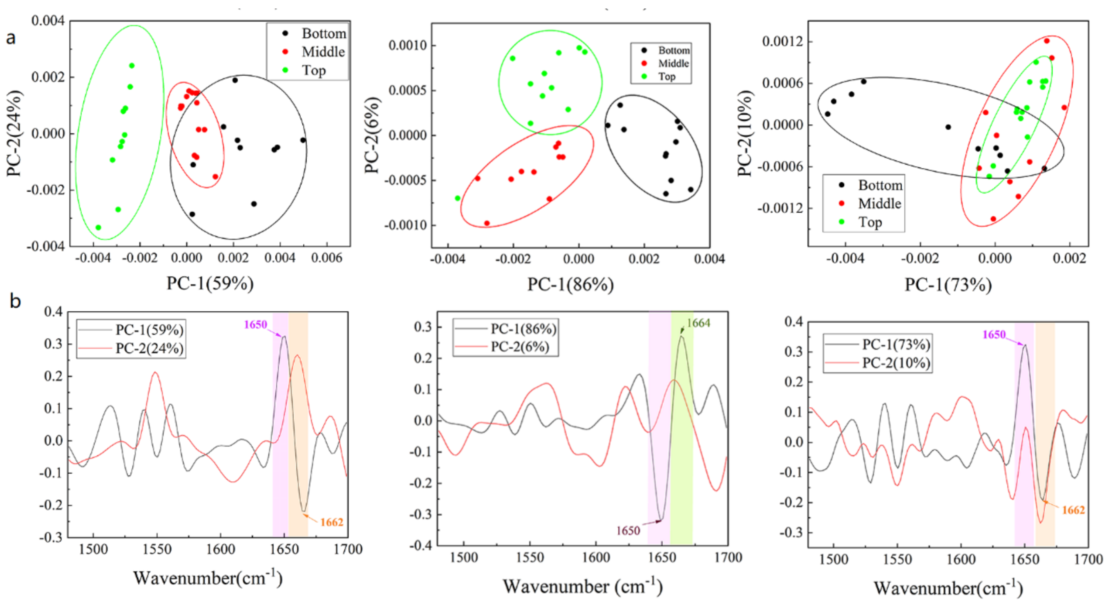

Figure S5. Score and loading plots of PCA at the different zones (left day 1, middle: day 3, right: day 5).

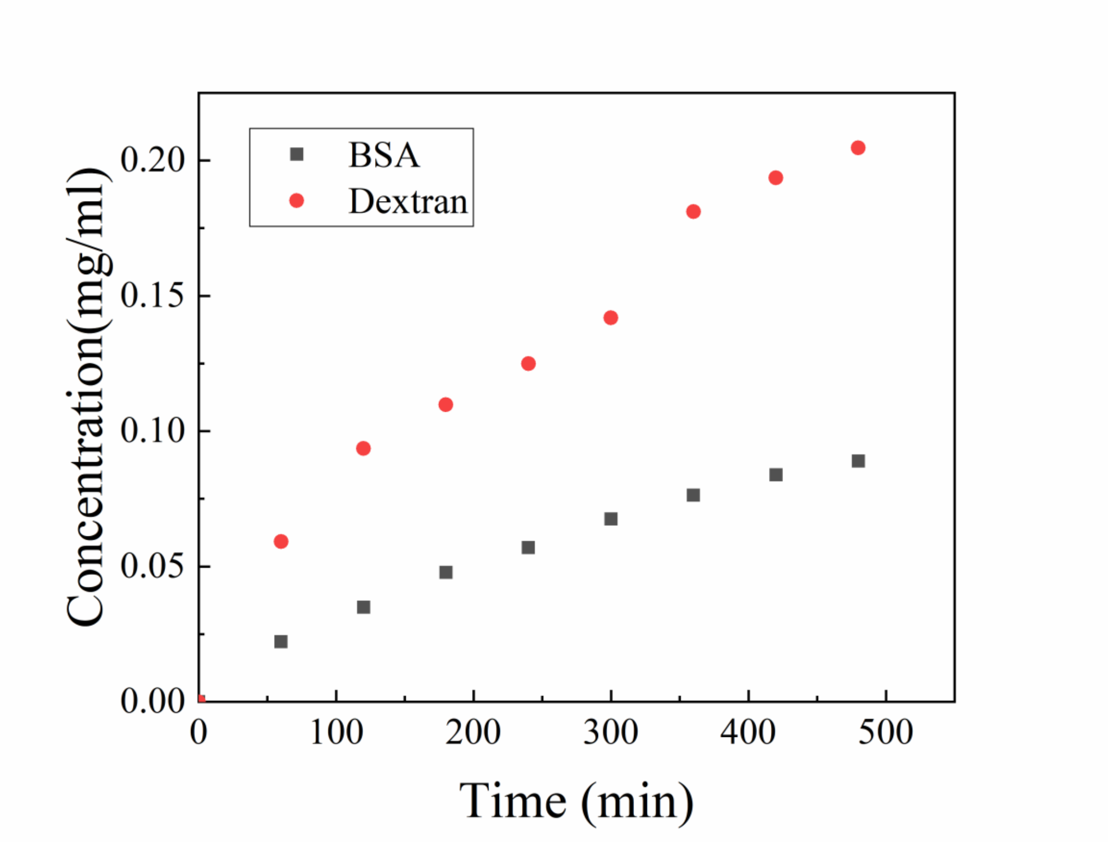

Figure S6. BSA and Dextran concentration change with time

Solute distribution in hydrogel

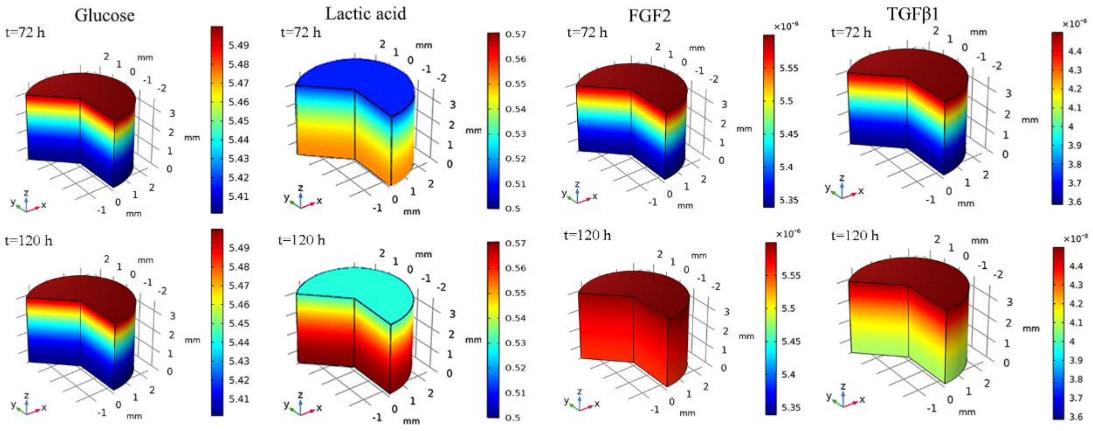

Figure S7. Solute distribution in hydrogel at 72 and 120 h

Table S1. Global parameters and variables for the COMSOL simulation.

Parameter	Value	Unit	Annotation
T	310	K	Temperature
$C_{g0,glucose}$	2.25	mol m^{-3}	Initial glucose conc. in gel
$C_{m0,glucose}$	5.5	mol m^{-3}	Initial glucose conc. in media
$C_{g,glucose}$	Variable	mol m^{-3}	Local glucose conc. in gel
$R_{glucose}$	1.108×10^{-5}	mol s^{-1} m^{-3}	Glucose uptake rate
$C_{g0, FGF2}$	2.8×10^{-6}	mol m^{-3}	Initial FGF2 conc. in gel
$C_{m0, FGF2}$	5.6×10^{-6}	mol m^{-3}	Initial FGF2 conc. in media
$C_{g, FGF2}$	Variable	mol m^{-3}	Real-time conc. of FGF2 in gel
$C_{g0, lacticacid}$	0	mol m^{-3}	Lactate conc. in gel
$C_{m0, lacticacid}$	0	mol m^{-3}	Lactate conc. in media
$C_{g, lacticacid}$	Variable	mol m^{-3}	local lactic acid conc. in gel
$C_{m, lacticacid}$	Variable	mol m^{-3}	local lactic acid conc. in media
$R_{lacticacid}$	1.108×10^{-5}	mol s^{-1} m^{-3}	Lactate production rate

Table S2. FTIR band assignment.

**Wavenumber/cm^{-1}**	**Functional group assignment**	**Biomolecule**
2970–2950	v_{as} CH_3	Lipid
2935–2915	v_{as} CH_2	Lipid
1742-1730	v(C=O) carbonyl	Phospholipids
1674–1662	Amide I, turn&bands	Protein
1650–1648	Amide I, vC=O (70-85%) and vC-N (10-20%) α-helix	Protein
1641–1633	Amide I, vC=O (70-85%) and vC-N (10-20%) β-sheet	Protein
1570–1530	Amide II, δN-H (40-60%), vC-N (18-40%) and vC-C (10%)	Protein
1397	v-COO-	Lipid
1379	δ_s CH_3	Lipid
1160	vC-O and δC-O-H	Carbohydrates
1099–1080	v_s PO_{2-}	DNA RNA phospholipids
1063	v_s PO_{2-},vC-C,vC-N	B-DNA
996	RNA stretch and bend ring of uracil	RNA
